# Motor-Evoked Potential Monitoring With Multi-train Electrical Stimulation During Thoracoabdominal Aortic Aneurysm Surgery: A Case Report

**DOI:** 10.7759/cureus.53872

**Published:** 2024-02-08

**Authors:** Takeo Yuno, Yusuke Nakade, Kenji Iino, Takumi Taniguchi, Hiroyasu Oe

**Affiliations:** 1 Clinical Laboratory, Kanazawa University Hospital, Kanazawa, JPN; 2 Thoracic, Cardiovascular, and General Surgery, Kanazawa University, Kanazawa, JPN; 3 Emergency and Critical Care Medicine, Graduate School of Medical Science, Kanazawa University, Kanazawa, JPN

**Keywords:** aortic aneurysm surgery, multi-train electrical stimulation, thoracoabdominal aortic aneurysm, motor evoked potential, intraoperative neurophysiological monitoring

## Abstract

Intraoperative motor-evoked potentials (MEPs) are measured for assessing motor function during surgery. MEP monitoring is often performed in thoracoabdominal aortic aneurysm (TAAA) surgery, but false positives are common and amplification methods are needed to obtain waveforms under severe conditions to assess proper spinal cord function. One method of amplitude amplification in transcranial-stimulated MEP monitoring is multitrain stimulation. There are few reports on multitrain-stimulated MEP monitoring for this surgery. A 57-year-old woman underwent open repair of the thoracoabdominal aorta due to a dissecting aortic aneurysm. After opening the chest, the aneurysm was incised proximally, and anastomosis with an artificial vessel was initiated. The lumbar artery leading to the Adam-Kiewicz artery was reconstructed at a body temperature of 25 °C. However, the single-train stimulation did not produce MEPs. When the measurement was switched to multitrain stimulation, MEPs were elicited in the lower extremity muscle groups and the waveforms were maintained until the end of the measurement. This case illustrates that MEP monitoring using multitrain stimulation during descending thoracic aortic aneurysm surgery can effectively elicit MEPs under challenging conditions, in which conventional single-train stimulation may be insufficient.

## Introduction

Intraoperative motor-evoked potential (MEP) monitoring has been used in many centers to avoid postoperative paraplegia due to ischemic spinal cord injury during thoracoabdominal aortic aneurysm (TAAA) surgery. However, false-positive results (decreased or absent waveforms without spinal cord ischemia) are common and their utility is unknown [1-3]. False-positive MEP frequently occurs during hypothermia and extracorporeal circulation for brain protection during surgery [2]. Therefore, to reduce false positives and improve the accuracy of MEP monitoring during surgery, amplitude amplification methods may be useful for deriving waveforms, even under conditions such as hypothermia and circulatory arrest, which are difficult to derive using conventional electrical stimulation methods [2]. Amplitude amplification methods for MEP include post-tetanic MEP, in which peripheral nerves are tetanus-stimulated for 3-5 s before transcranial stimulation [4], and multitrain stimulation MEP, in which pulse trains are repeated at low frequencies [5].

Although multitrain stimulation has been reported in spinal surgery [5], there are few reports on its use in thoracoabdominal aortic artificial vessel replacement. This study reports a case in which MEP was not derived by conventional single-train stimulation during thoracoabdominal descending aortic aneurysm surgery but by multitrain stimulation.

## Case presentation

A 57-year-old woman with Marfan syndrome underwent open descending aortic repair of a chronic dissecting aortic aneurysm because the maximum aneurysm diameter increased to 58 mm during follow-up. Seven years previously, she had undergone aortic root and total aortic arch replacement for Stanford Type A acute aortic dissection utilizing a 25-mm aortic-valved graft (St. Jude Medical, Inc., Little Canada, Minnesota, US), and a 26-mm 4-branched J-Graft (Japan Lifeline, Tokyo, Japan). A spinal drainage catheter was inserted the day before surgery to safeguard the spinal cord, and MEP monitoring was conducted during the surgery.

**Figure 1:**
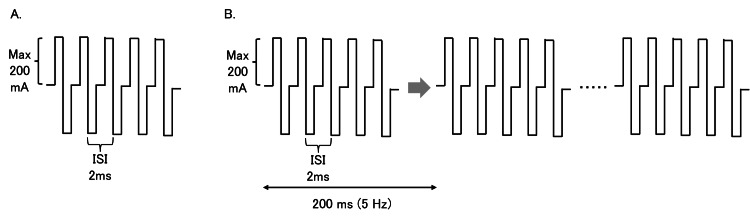
Overview of single-train and multi-train stimulation In the biphasic stimulation, the first stimulation was immediately followed by a second counterphasic stimulation. In the single-train stimulation method, biphasic square-wave electrical stimulation (5-pulse train, 2 ms stimulation interval, 0.5 ms duration, and 200 mA maximum intensity) was performed once (A). The multitrain stimulation method was performed n times at 5 Hz, which was performed two to three times in this case (B). ISI: interstimulus interval

MEP monitoring was performed using the Neuromaster G1 (Nihon Kohden, Tokyo, Japan). Electrical stimulation was performed by transcranial stimulation using corkscrew electrodes placed at C3 and C4 of the international 10-20 EEG system for electrode placement [6]. Single-train stimulation consisted of five trains of 0.5 ms stimulation time, 2 ms stimulation interval, and 200 ms recording time, with the phase set to biphasic (Figure 1A). For multitrain stimulation, as reported in previous studies [5,7,8], either two or three 5-train stimulations were administered at 5 Hz, featuring a biphasic phase, a stimulus duration of 0.5 ms, an interval between stimuli of 2 ms, and a total recording duration of 200 ms (Figure 1B). The stimulation intensity was set to a maximum of 200 mA with maximal supramaximal stimulation in the lower extremity muscle groups. Paired needle electrodes were placed on the abductor hallucis (AH), tibialis anterior (TA), and gastrocnemius (GA) as the monitored muscles. The abductor pollicis brevis (APB) was used as a control muscle to monitor anesthesia and other systemic conditions. The alarm point was defined as the disappearance of the MEP waveform based on a previous report [9]. Anesthesia was induced during MEP monitoring using total intravenous anesthesia with propofol. No muscle relaxants were used during MEP monitoring.

**Figure 2:**
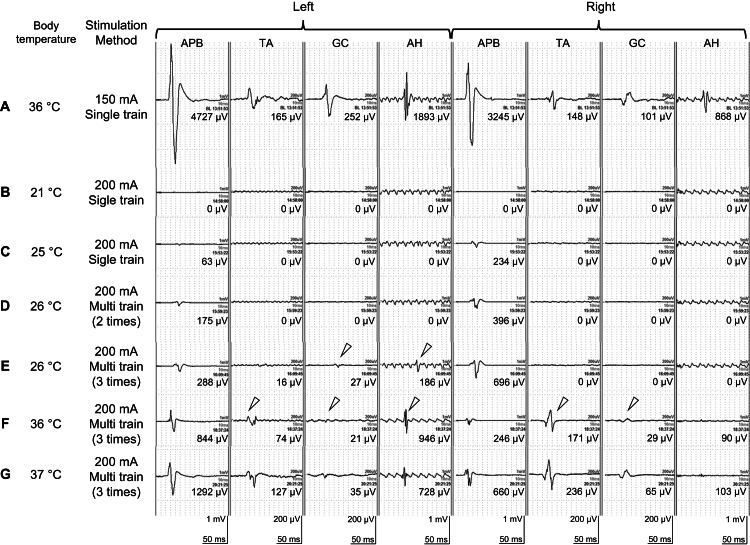
Representative waveforms of motor-evoked potentials in the present case A. Baseline (after chest opening, before start of cooling), B. During hypothermic circulatory arrest, C. After reconstruction of the lumbar artery with Hemashield grafts (single-train stimulation), D. After reconstruction of the lumbar artery with Hemashield grafts (multi-train stimulation, two times), E. After reconstruction of the lumbar artery with Hemashield grafts (multi-train stimulation, three times), F. After anastomosis of the central side of Hemashield graft to Cocelli and Triplex grafts, G. At final measurement before the closed wound. MEP, motor-evoked potential; SEP, somatosensory-evoked potential; APB, abductor pollicis brevis; TA, tibialis anterior; GA, gastrocnemius; AH, abductor hallucis

A straight incision was made from the axilla to the left umbilical area, preserving the thoracodorsal artery and the surrounding muscles. The chest cavity was accessed through the sixth intercostal space with the ribs divided along the incision line. The diaphragm was circumferentially cut, leaving some muscle edges at costal insertion. The abdominal aorta was exposed through the retroperitoneal cavity by using a wishbone retractor, revealing it from the descending aorta to the bilateral common iliac arteries. At this stage, MEP was measured using a single 150 mA stimulus, and the resulting waveform was established as the baseline (Figure 2A). Cardiopulmonary bypass (CPB) was initiated with an 8-mm synthetic graft anastomosed to the left axillary artery, and cannulation was performed at the left common femoral artery and vein. Additionally, a left-heart venting cannula was inserted through the left pulmonary vein. During cooling, the patient experienced ventricular fibrillation. Preoperative MRI revealed that the Adam-Kiewicz artery (AKA) originated from the first lumbar artery of the false lumen and the third lumbar artery of the true lumen. When tympanic and bladder temperatures reached 21°C, the descending aorta was clamped proximal to the suspected AKA to maintain femoral artery perfusion. The thoracic descending aorta was opened, and patent intercostal arteries (ICAs) were oversewn during upper-body circulatory arrest. Open proximal anastomosis to the J-graft was performed in the previous surgery by using a 26-mm 1-branched Triplex (Terumo Corp., Tokyo, Japan) with retrograde cerebral perfusion. Simultaneously, the stimulation intensity was increased to 200 mA, and MEP measurements were obtained, resulting in a loss of response in all the muscles (Figure 2B). The proximal clamp was applied distally to the side branch of the Triplex, restoring blood flow through the side branch of the Triplex and femoral artery. Subsequently, the aortic clamp was shifted from the descending aorta to the abdominal aorta. Balloon occlusion catheters were placed in the first and third lumbar arteries to limit back bleeding. Visceral arteries were cannulated and perfused at a total rate of 800-1000 ml/min. The first and third lumbar arteries were individually anastomosed and perfused using two 10-mm Hemashield grafts (Getinge, Gothenburg, Sweden). At this point, at a body temperature of 25°C, no MEP was observed in any of the lower-extremity muscle groups after single-train stimulation at 200 mA (Figure 2C).

Therefore, when we switched to multitrain stimulation, no MEPs were observed in the lower limb muscle groups after the two stimulations (Figure 2D). However, when the number of stimulations was increased to three, small amplitude, reproducible MEP waveforms were observed in the left GC and AH (Figure 2E). Visceral arteries were individually reconstructed using a 4-vessel branch 24-mm Coselli graft (Vascutek Terumo, Renfrewshire, UK) and anastomosed to a Triplex graft in an end-to-end manner. Finally, two Hemashield grafts anastomosed to the lumbar arteries were individually connected to the Cocelli and Triplex grafts in an end-to-side manner.

MEP was measured following the reattachment of the lumbar arteries, and MEP waveforms were confirmed in the bilateral TA, GA, and left AH (Figure 2F). After weaning from CPB and achieving minute hemostasis, the diaphragm was reapproximated, and the wound was closed. MEP waveforms were obtained for the bilateral APB, TA, GC, and AH at the final measurement, before closing the wound (Figure 2G). The patient demonstrated movement in both lower extremities on the second postoperative day. To date, no paralysis of the lower extremities has been observed.

## Discussion

In the present case, multi-train stimulation was used during MEP monitoring in TAAA surgery, which could have led to a more appropriate evaluation of the spinal cord function than conventional single-train stimulation. The accuracy of MEP monitoring in this surgery was generally low due to the frequent false positives due to various factors. This case demonstrates that multi-train stimulated MEP monitoring can potentially reduce false positives.

In our case, after reconstruction of the lumbar artery leading to the Adam-Kiewicz artery at a body temperature of 25°C, MEPs in the lower extremities could be detected with three multi-train stimulations, but not with normal single-train stimulation. The evaluation of MEPs after Adam-Kiewicz artery reconstruction is important in this surgery. Specifically, the fact that MEPs can be detected with multi-train stimulation at this stage has great clinical significance, because it indicates that reconstruction is appropriate and that there is no spinal cord ischemia. In addition, MEPs were obtained from lower-extremity muscle groups with multi-train stimulation at the end of monitoring, and no postoperative paraplegia was observed. This observation indicates that the MEPs from multi-train stimulation in this surgery also reflect the patient's spinal cord function and that using this method may improve the accuracy of MEP monitoring in TAAA surgery.

Aortic arterial replacement with hypothermic circulatory arrest, as in this case, often makes it difficult to derive MEP, even in the absence of spinal cord ischemia. Therefore, increasing the motor response elicited by transcranial stimulation is necessary to detect spinal cord ischemia adequately. In addition to multi-train stimulation, this augmentation method includes post-tetanic MEP, which involves tetanus stimulation of the peripheral nerves for 3-5 seconds before transcranial stimulation. In the present study, we selected a multi-train stimulation MEP based on the simplicity of the setting and reports of amplitude augmentation during spinal surgery [5,7]. In the multi-train stimulation MEP in the present case, train stimulation was performed three times at 5 Hz. However, in a previous report [7], a 5-train stimulation was repeated 2-7 times at a frequency of 2-10 Hz. Therefore, MEP can still be elicited in patients who do not respond to the three trains used in this study by adjusting the number of trains and the stimulation frequency settings. This adjustment allows for monitoring even under more severe conditions. However, when modifying stimulation conditions, such as using multi-train stimulation during monitoring, caution must be exercised in routine monitoring, as it becomes challenging to objectively assess the extent of deviation from the baseline.

In contrast, in TAAA surgery using hypothermic circulatory arrest, spinal cord ischemia is assessed by the presence or absence of waveforms and not by the MEP amplitude ratio [9,10]. Therefore, amplitude amplification methods, such as multi-train stimulation, can be adopted during surgery, as in the present case, in which hypothermic circulatory arrest or aortic blockade caused MEP loss.

Problems with multi-train stimulation MEP monitoring have been reported to increase body movements and bites associated with stimulation [11]. In the present case, the bite block with gauze did not cause bite wounds, and the body movements were not large enough to affect the surgical manipulation. This decrease in movement could be attributed to the patient being hypothermic or post-restorative at the time of the multi-train stimulation.

This is a report of a single case at a single site, and because only multi-train stimulation was used in the final measurements, it is possible that MEPs were also detected during single-train stimulation. In addition, the conditions of multi-train stimulation, presence or absence of preoperative patient paralysis, anesthetic conditions, duration of artificial cardiopulmonary or circulatory arrest, minimum body temperature, and type of muscle being monitored may have affected the results. Therefore, further investigation is needed to correlate the monitoring results of multi-train stimulation with postoperative paraplegia and to reduce false positives. However, this case report demonstrated that multi-train-stimulated MEP monitoring enables the detection of MEPs in situations previously undetectable by single-train stimulated MEPs.

## Conclusions

Here, we report a case of MEP monitoring using multi-train stimulation in a patient undergoing surgery for a thoracoabdominal aortic aneurysm. In the present case, MEPs of the lower extremity muscles could be derived by adapting to multi-train stimulation, even after hypothermic circulatory arrest, which could not be derived by single-train stimulation. Thus, multi-train stimulation can potentially reduce false positives, which are a problem in MEP monitoring during surgery. This advantage is clinically significant because it improves the accuracy of MEP monitoring and enables earlier and more accurate detection of intraoperative spinal cord ischemia, thereby reducing the risk of postoperative paraplegia. Further studies are needed to clarify the clinical efficacy of multi-train stimulation for intraoperative MEP monitoring.
